# Real-World Efficacy of Minimally Invasive Revascularization in Diabetic Foot Ischemia: Impact of Device Selection and Lesion-Specific Factors

**DOI:** 10.3390/biomedicines13061384

**Published:** 2025-06-05

**Authors:** Yue Lin, Fanzhen Lv, Yulong Huang, Gang Chen, Shichai Hong, Xiang Hong, Xinsheng Xie, Weifeng Lu, Weiguo Fu

**Affiliations:** 1 Department of Vascular Surgery, Xiamen Branch, Zhongshan Hospital, Fudan University, Xiamen 361015, China; lin.yue@zsxmhospital.com (Y.L.);; 2 Department of Vascular Surgery, Zhongshan Hospital, Fudan University, Shanghai 200032, China

**Keywords:** revascularization, diabetic foot ischemia, patency, CLTI, PAD

## Abstract

**Objectives**: The objective of this study was to evaluate the real-world efficacy of minimally invasive revascularization in diabetic foot ischemia, focusing on novel insights into device selection and lesion-specific predictors. **Methods**: This retrospective study included 98 patients (101 limbs) undergoing endovascular/hybrid interventions. The primary endpoints were 1- and 2-year primary patency and freedom from clinically driven target lesion revascularization (CD-TLR). Multivariate Cox regression identified restenosis predictors, with subgroup analysis comparing drug-coated devices (DCDs) versus conventional strategies in chronic limb-threatening ischemia (CLTI). **Results**: The cohort (mean age 72.1 ± 8.9 years) comprised 51% CLTI limbs (28.5% with tissue loss). The overall 1-year primary patency was 75.6%, declining to 67.6% after 2 years. The rates of freedom from CD-TLR were 87.4% after 1 year and 74.8% after 2 years. CLTI was associated with significantly reduced 1-year (66.5% vs. 84.9%) and 2-year primary patency (56.3% vs. 80.1%; log-rank *p* = 0.026) compared to non-CLTI. Multivariate analysis identified CLTI as an independent predictor of restenosis (HR 3.375, 95%CI 1.267–8.990, *p* = 0.015). Although DCDs did not improve 2-year primary patency in CLTI (58.5% vs. 57.3%, *p* = 0.768), they demonstrated superior 2-year CD-TLR-free survival (78.5% vs. 54.6%, *p* = 0.048). The total complication rate was 5.9%, with no significant difference between CLTI and non-CLTI groups (11.5% vs. 0%, *p* = 0.057). **Conclusions**: This study highlights CLTI’s impact on revascularization durability and the clinical benefits of DCDs in reducing reinterventions, offering evidence-based insights for tailored device selection despite retrospective limitations.

## 1. Introduction

Revascularization strategies for diabetic foot ischemia, especially in critical limb-threatening ischemia (CLTI), remain a topic of ongoing debate. The discrepancy between anatomical success and clinical outcomes in CLTI necessitates mechanistic elucidation [1,2,3,4]. Although drug-coated devices (DCDs) enhance femoropopliteal patency rates, diabetes-specific microvascular dysfunction may restrict their clinical effectiveness in certain CLTI subgroups [5,6,7,8]. Additionally, atherosclerosis plaques in the femoropopliteal segment are characterized by advanced calcification, which is associated with medial tunica calcinosis occurring at relatively young ages [9]. Previous studies have highlighted the expanding application of endovascular treatment in diabetic patients [10]. However, these patients demonstrate reduced primary patency rates following endovascular revascularization of the lower extremities, with particularly unfavorable outcomes observed in cases of poor perioperative glycemic control [11,12].

This real-world study aims to explore (1) the relative impact of CLTI on midterm revascularization durability in diabetic ischemia and (2) interactions between device selection and key clinical characteristics. Through multivariate modeling and subgroup analysis, we provide evidence-based insights to refine personalized revascularization frameworks.

## 2. Materials and Methods

### 2.1. Study Design and Data Collection

This single-center retrospective cohort study analyzed consecutive patients with diabetic foot ischemia undergoing endovascular/hybrid revascularization between January 2018 and December 2024. Data were extracted from a prospectively maintained institutional vascular registry, including baseline characteristics (age, sex, smoking status, hypertension, hyperlipidemia, chronic renal insufficiency, coronary artery disease), clinical parameters (pre/post-procedural ankle brachial index [ABI], lesion length, Rutherford category), and lesion features (stenosis/occlusion type, chronic total occlusion [CTO] status, TransAtlantic Inter-Society Consensus II [TASC II] classification [13], bifurcation involvement via Medina criteria [14], and calcification implied by debulking device use). Below-the-knee (BTK) outflow was defined by the number of patent tibial/fibular arteries (1-vessel, 2-vessel, or 3-vessel). Vascular anatomy and lesion complexity were evaluated using computed tomography angiography (CTA) and intraprocedural digital subtraction angiography (DSA), with classification adhering to contemporary peripheral artery disease (PAD) guidelines.

The study protocol adhered to STROBE guidelines and was approved by the institutional ethics committee (B2025-018).

### 2.2. Procedure Details

**All patients received dual antiplatelet therapy (clopidogrel 75 mg + aspirin 100 mg daily) and statin therapy (atorvastatin 20 mg daily) for at least 7 days prior to the intervention**. Postoperatively, dual antiplatelet therapy (aspirin + clopidogrel) was continued for 6 months, after which patients were switched to long-term monotherapy with either aspirin or clopidogrel. Statin therapy (atorvastatin 20 mg daily) was maintained long-term. Intravenous heparin (70 U/kg) was administered intraoperatively. Endovascular procedures were performed under local anesthesia, while hybrid surgeries required general anesthesia. For endovascular access, contralateral common femoral artery (CFA) puncture was used for femoral artery lesions, ipsilateral sequential access for distal superficial femoral artery combined with below-the-knee (BTK) lesions, and retrograde puncture of patent distal segments for chronic occlusions. Hybrid procedures consisted of open-CFA endarterectomy with patch angioplasty, followed by endovascular treatment of proximal (e.g., iliac) or distal (e.g., superficial femoral artery or BTK) lesions via direct patch puncture. Lesion preparation included angiography for anatomical assessment, atherectomy for calcified stenosis, and balloon angioplasty with prolonged inflation for residual stenosis >30%. Stent selection (bare-metal stents vs. drug-eluting stents) and drug-coated balloon (DCB) use were determined intraoperatively based on lesion characteristics, operator preference, and patient affordability. Self-expanding stents were deployed for flow-limiting dissections, while DCBs were prioritized for long or restenotic lesions when feasible. In CLTI cases, BTK outflow management was determined intraoperatively based on operator judgment. Post-procedural care included femoral access closure with vascular devices, 24-hour monitoring, and thrombectomy/stent recovery for complications. This approach balanced lesion complexity, operator discretion, and patient-specific factors to optimize revascularization outcomes.

### 2.3. Outcomes and Follow-Up Evaluation

The study evaluated three primary endpoints: 1- and 2-year primary patency, freedom from clinically driven target lesion revascularization (CD-TLR), and complications. Primary patency was defined as continuous stent patency without restenosis (>50% luminal narrowing) on angiography/CTA. CD-TLR was triggered by recurrent ischemia symptoms (e.g., rest pain, tissue loss) combined with duplex/CTA evidence of ≥50% stenosis within 5 mm of the treated segment. Major amputation was defined as any amputation above the ankle.

### 2.4. Follow-Up Protocol

Immediate post-procedure protocol: clinical exam, ABI measurement, and angiography to confirm technical success (residual stenosis <30%).

Routine follow-up: At 1, 3, 6, and 12 months, including clinical assessment (symptom resolution, wound healing); duplex ultrasound or CTA for restenosis detection; and ABI measurement to assess hemodynamic improvement. Long-term follow-up: annual clinical and imaging evaluations for up to 24 months.

### 2.5. Complication Tracking

Adverse events were recorded for 30 days post-procedure, including major adverse limb events (MALEs), such as amputation, CD-TLR, or limb loss, or procedural complications, such as stent fracture, hematoma, or a cerebrovascular accident.

### 2.6. Statistical Analysis

Data were analyzed using SPSS (version 26.0). Continuous variables were reported as mean ± SD or median (IQR), while categorical variables were presented as counts (%). Group differences between non-CLTI and CLTI patients were compared using independent t-tests for continuous variables and chi-squared/Fisher’s exact tests for categorical variables. Survival outcomes, including primary patency and freedom from clinically driven target lesion revascularization (CD-TLR), were estimated via Kaplan–Meier curves and compared using log-rank tests. Multivariate Cox regression was performed to identify predictors of restenosis, adjusting for CLTI status, lesion complexity (TASC II classification), CTO status, BTK outflow quality, and device type (drug-coated vs. conventional). Subgroup analyses compared outcomes between the CLTI and non-CLTI subgroups and between DCDs and conventional strategies. Complications were evaluated using chi-squared/Fisher’s exact tests. Statistical significance was set at *p* < 0.05.

## 3. Results

### 3.1. Baseline Characteristics and Overall Outcomes

**Figure 1:**
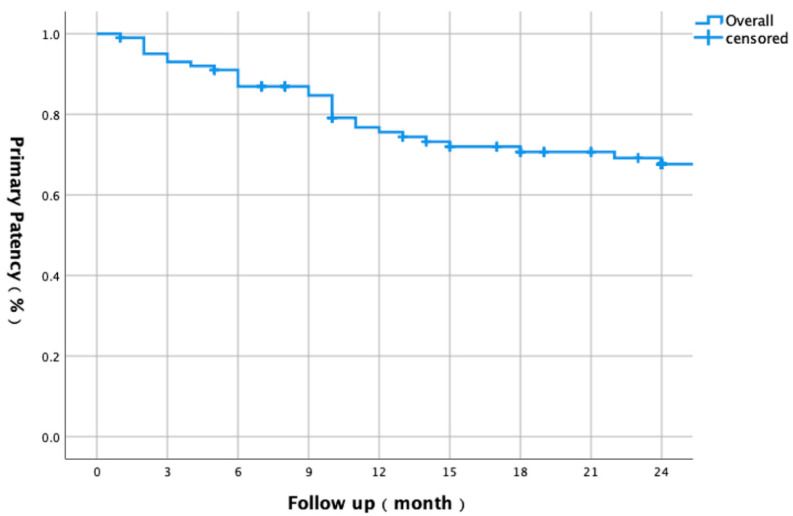
Kaplan–Meier curve showing two-year primary patency rates for all lesions (*n* = 101) in patients with diabetic foot ischemia. Primary patency was 75.6% at 1 year and 67.6% at 2 years.

**Figure 2:**
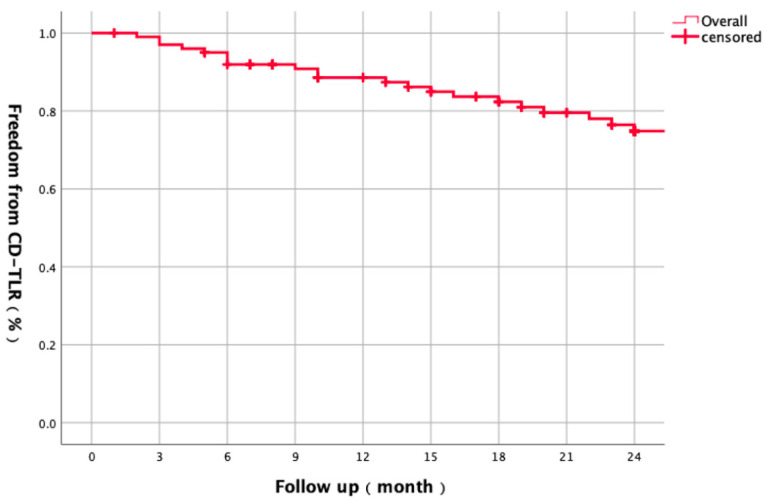
Kaplan–Meier curve illustrating two-year freedom from clinically driven target lesion revascularization (CD-TLR) for all lesions (*n* = 101). Freedom from CD-TLR was 87.4% at 1 year and 74.8% at 2 years.

This study included 98 patients (101 limbs) with diabetic foot ischemia (mean age 72.1 ± 8.9 years; 75.5% male). Critical limb-threatening ischemia (CLTI) was present in 51% of limbs (28.5% with tissue loss). Lesions averaged 26.1 ± 9.5 cm in length, with 91.9% classified as TASC C/D. The post-procedural ankle–brachial index (ABI) significantly improved from 0.43 ± 1.40 to 0.81 ± 0.16 (*p* < 0.001) (Table 1). The overall 1-year primary patency was 75.6%, declining to 67.6% after 2 years (Figure 1). Freedom from CD-TLR was 87.4% after 1 year and 74.8% after 2 years (Figure 2).

### 3.2. Multivariate Cox Regression

CLTI independently predicted restenosis/occlusion (HR = 3.375, 95%CI 1.267–8.990, *p* = 0.015), whereas drug-coated devices (DCDs) showed no significant protective effect (HR = 0.525, *p* = 0.218). Lesion length per cm increment trended toward increased risk (HR = 1.038, *p* = 0.113) (Table 2). However, comorbidities such as hypertension (HR = 0.979, *p* = 0.972) and coronary heart disease (HR = 1.915, *p* = 0.199) did not significantly influence restenosis risk in the multivariate analysis, suggesting that CLTI status was the dominant predictor.

### 3.3. CLTI vs. Non-CLTI Comparisons

**Figure 3:**
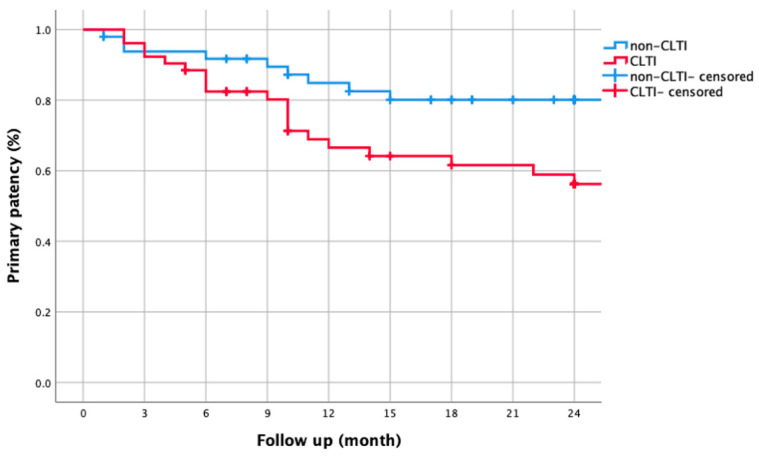
Kaplan–Meier curve comparing two-year primary patency rates between non-CLTI and CLTI groups. Non-CLTI patients had significantly higher patency (84.9% vs. 66.5% at 1 year; 80.1% vs. 56.3% at 2 years, log-rank *p* = 0.026).

**Figure 4:**
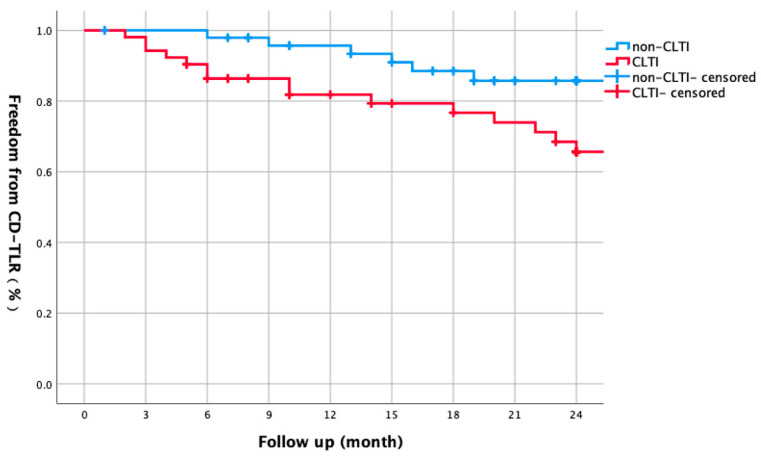
Kaplan–Meier curve comparing two-year freedom from CD-TLR between non-CLTI and CLTI groups. Non-CLTI patients had higher CD-TLR-free survival (93.4% vs. 79.3% at 1 year; 85.7% vs. 65.6% at 2 years, log-rank *p* = 0.035).

CLTI patients had higher hypertension prevalence (90.4% vs. 73.5%, *p* = 0.020) and marginally longer lesions (27.8 ± 9.5 cm vs. 24.3 ± 9.2 cm, *p* = 0.060) (Table 3). The CLTI group exhibited lower 1-year primary patency (66.5% vs. 84.9%, log-rank *p* = 0.026), with the gap widening after 2 years (56.3% vs. 80.1%; Figure 3). CD-TLR-free survival was significantly reduced in CLTI—79.3% vs. 93.4% after 1 year (*p* = 0.035), declining to 65.6% vs. 85.7% after 2 years (Figure 4).

### 3.4. DCD Subgroup Analysis

**Figure 5:**
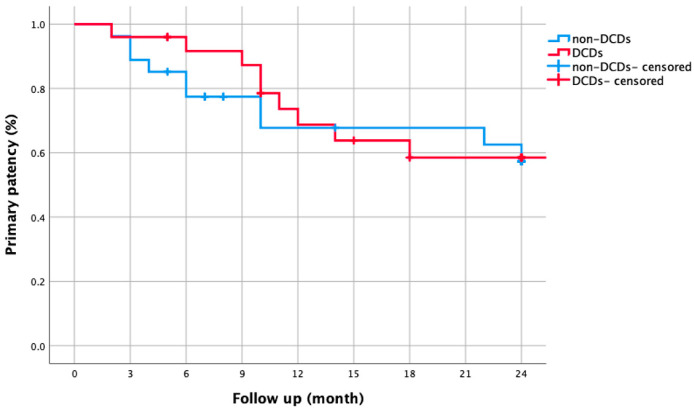
Subgroup analysis: Kaplan–Meier curve comparing two-year primary patency rates in CLTI patients with versus without drug-coated devices (DCDs). Patency rates were similar (68.7% vs. 67.8% at 1 year; 58.5% vs. 57.3% at 2 years, log-rank *p* = 0.768).

**Figure 6:**
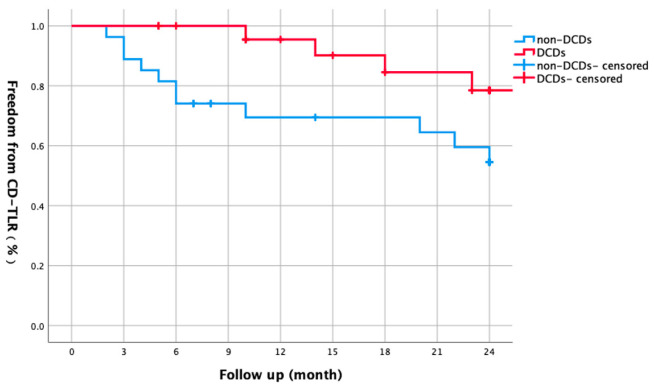
Subgroup analysis: Kaplan–Meier curve comparing two-year freedom from CD-TLR in CLTI patients with versus without DCDs. DCD use was associated with improved CD-TLR-free survival (90.2% vs. 69.4% at 1 year; 78.5% vs. 54.6% at 2 years, log-rank *p* = 0.048).

Among CLTI patients, DCD use did not improve 1-year (68.7% vs. 67.8%) or 2-year patency (58.5% vs. 57.3%, *p* = 0.768; Figure 5). However, DCDs significantly enhanced CD-TLR-free survival—90.2% vs. 69.4% after 1 year (*p* = 0.048), remaining at 78.5% vs. 54.6% after 2 years (Figure 6).

### 3.5. Complications

The total complication rate was 5.9% (6/101), including four major amputations, one access-site hematoma, and one perioperative cerebrovascular accident. Complications occurred more frequently in CLTI (11.5%, 6/52) than non-CLTI (0%, 0/49) patients, but the difference was not statistically significant (*p* = 0.057).

## 4. Discussion

This retrospective study meticulously examined the outcomes of minimally invasive revascularization in patients with diabetic foot ischemia, with a particular emphasis on the influence of device selection and lesion-specific factors. The study cohort comprised 98 patients (101 limbs) who underwent endovascular or hybrid interventions. Our findings underscore that chronic limb-threatening ischemia (CLTI) significantly compromises the durability of revascularization, with an independent prediction of restenosis (HR = 3.375, *p* = 0.015). Although drug-coated devices (DCDs) did not enhance primary patency in CLTI patients, they demonstrated a notable reduction in clinically driven target lesion revascularization (CD-TLR), with a 2-year CD-TLR-free survival rate of 78.5% compared to 54.6% in those treated with conventional devices (*p* = 0.048). These results highlight the complexity of managing diabetic foot ischemia and the need for tailored revascularization strategies.

Our study revealed a stark contrast in clinical outcomes between CLTI and non-CLTI patients. The 1- and 2-year primary patency rates were significantly lower in CLTI patients (66.5% vs. 84.9% at 1 year and 56.3% vs. 80.1% at 2 years, log-rank *p* = 0.026), indicating that CLTI is a robust predictor of restenosis. This aligns with previous research [6], which identified lesion morphology and calcification as dominant predictors of restenosis, even in non-diabetic cohorts. The CAPSICUM study confirms that CLTI is a risk factor affecting patency rate [15]. However, in diabetic CLTI, the interplay of hyperglycemia-induced endothelial dysfunction and oxidative stress likely exacerbates neointimal hyperplasia, as evidenced by our multivariate analysis. Furthermore, the higher complication rates observed in CLTI patients (11.5% vs. 0% in non-CLTI, *p* = 0.057) underscore the therapeutic challenges associated with this condition. These findings collectively highlight the need for more aggressive and tailored interventions in CLTI patients to improve long-term outcomes.

The management of ischemic diabetic foot remains a formidable challenge, particularly in the context of CLTI. Recent studies have consistently reported suboptimal outcomes in this patient population. For instance, a meta-analysis by Rizzo et al. [9] demonstrated that diabetes accelerates arterial restenosis through metabolic dysregulation, with restenosis rates reaching up to 60% within 2 years. Similarly, a study by Pearce et al. [10] highlighted that diabetic patients with CLTI frequently require multilevel revascularization, emphasizing the complexity of treating concurrent tibial disease. Our study’s findings of 1-year and 2-year primary patency rates of 66.5% and 56.3%, respectively, in CLTI patients further illustrate the ongoing challenges. Despite advancements in interventional devices, such as drug-coated balloons (DCBs) and drug-eluting stents (DESs), their impact on long-term outcomes remains controversial. For example, the IN.PACT SFA trial reported promising results with DCBs, but real-world studies have shown variable efficacy, particularly in diabetic CLTI patients [6,7]. This underscores the need for more robust and tailored therapeutic strategies to address the multifaceted pathophysiology of diabetic foot ischemia.

The efficacy of DCDs, including DCBs and DESs, in treating CLTI remains a topic of intense debate [15,16,17,18,19,20]. Our study demonstrated that while DCDs did not significantly improve primary patency in CLTI patients, they significantly enhanced CD-TLR-free survival (78.5% vs. 54.6% at 2 years, *p* = 0.048). This finding is consistent with several other studies. For instance, the ILLUMENATE trial [21,22] showed that DCBs could reduce the need for reintervention in CLTI patients, although anatomical patency was not consistently improved. Similarly, the EMINENT Randomized Trial [23] reported that DES provided better long-term outcomes compared to bare-metal stents, but the benefits were not uniform across all patient subgroups. Other studies, such as the RANGER trial [24,25] and the LEVANT 2 trial [26,27], have also explored the potential advantages of DCDs in CLTI, with mixed results. Our study’s strength lies in its comprehensive evaluation of both anatomical and clinical outcomes, providing nuanced insights into the potential benefits of DCDs in diabetic foot ischemia. These findings suggest that DCDs may play a crucial role in stabilizing plaque morphology and reducing embolic burden, thereby mitigating symptom-driven reinterventions, even without sustained anatomical patency.

Advanced diabetes can lead to a ‘desert foot’ phenomenon, characterized by severe distal microvascular disease with no identifiable revascularization targets in the foot. This condition, often seen in CLTI patients, contributes to poor long-term outcomes and reduced patency rates, as revascularization efforts may fail to restore adequate perfusion to the distal tissues. This underscores the need for comprehensive diabetic management and early intervention to prevent progression to this stage.

This study has several limitations inherent to its retrospective design. Selection bias may have influenced the choice of revascularization strategies and device selection, despite efforts to minimize confounding factors during data collection. The relatively small sample size and single-center nature of the study may limit the generalizability of our findings. Additionally, the follow-up period was restricted to 2 years, which may not fully capture the long-term durability of revascularization. Future prospective, multicenter studies with larger cohorts and extended follow-up are essential to validate these findings and refine treatment strategies.

## 5. Conclusions

CLTI substantially compromises revascularization durability in diabetic foot ischemia. The dissociation between the anatomical patency and clinical benefits of DCDs underscores the importance of lesion-specific device selection strategies.

## Figures and Tables

**Table 1:** Baseline characteristics and outcomes for patients with peripheral arterial disease (*N* = 98, legs = 101).

Variables	*N* = 98, legs = 101
Age (years)	72.11 ± 8.90
Male [*n* (%)]	74 (75.5%)
Follow-up duration (months)	16.57 ± 10.25
Comorbidities	
Hypertension [*n*(%)]	83 (84.7%)
Smoking [*n*(%)]	49 (50.0%)
Hyperlipidemia [*n*(%)]	72 (73.5%)
Chronic renal insufficiency [*n*(%)]	10 (10.2%)
Coronary heart disease [*n*(%)]	21 (21.4%)
Clinical Parameters	
ABI * pre-procedure	0.43 ± 1.40
ABI * post-procedure	0.81 ± 0.16
Lesion length (cm)	26.11 ± 9.47
Claudication [*n*(%)]	49 (49%)
CLTI * [*n*(%)]	52 (51%)
Rest pain	23 (22.5%)
Tissue loss	29 (28.5%)
TASC * Classification [*n*(%)]	
A + B	10 (10.1%)
C + D	91 (91.9%)
Lesion Characteristics	
CTO * lesions [*n*(%)]	88 (87.1%)
Stenosis [*n*(%)]	12 (11.9%)
Runoff Grade [*n*(%)]	
1	43 (42.6%)
2	29 (28.7%)
3	28 (27.7%)
Procedural Details	
Endovascular intervention [*n*(%)]	96 (95.0%)
Hybrid procedure * [*n*(%)]	5 (5.0%)
Concurrent BTK * outflow treatment [*n*(%)]	31 (30.7%)
Stent procedure [*n*(%)]	101 (100%)
Debulking device use [*n*(%)]	2 (2.0%)
DCDs * use [*n*(%)]	40 (39.6%)
Complications	
Total complications [*n*(%)]	6 (5.9%)
MALE *	4 (4.0%)
Hematoma	1 (1.0%)
Cerebrovascular accident	1 (1.0%)

* Notes: ABI = ankle–brachial index; CLTI = critical limb-threatening ischemia; hybrid procedure: this involved CFA endarterectomy with/without patch angioplasty and endovascular treatment of proximal or distal lesions. BTK = below-the-knee; DCDs = drug-coated devices; TASC = Trans-Atlantic Inter-Society Consensus; MALE = major amputation limb events.

**Table 2:** Independent risk factors associated with restenosis/occlusion using multivariate Cox regression analysis.

Variable	B	SE	Wald	df	*p*-Value	HR	95% CI
Gender	0.109	0.564	0.037	1	0.847	1.115	0.369–3.365
Smoking history	0.469	0.506	0.86	1	0.354	1.599	0.593–4.314
Hypertension	−0.021	0.594	0.001	1	0.972	0.979	0.305–3.138
Hyperlipidemia	0.056	0.439	0.016	1	0.898	1.058	0.448–2.499
Coronary heart disease	0.65	0.506	1.646	1	0.199	1.915	0.710–5.164
Renal insufficiency	0.811	0.624	1.69	1	0.194	2.25	0.662–7.644
CLTI	1.217	0.5	5.924	1	0.015 *	3.375	1.267–8.990
Procedural details	−0.261	0.852	0.094	1	0.76	0.771	0.145–4.091
DCD usage	−0.645	0.524	1.515	1	0.218	0.525	0.188–1.465
Concurrent treatment of BTK outflow	0.228	0.461	0.244	1	0.621	1.256	0.509–3.097
Outflow status	0.265	0.457	0.336	1	0.562	1.303	0.532–3.190
Type of lesions (stenosis or occlusion)	0.263	0.89	0.087	1	0.768	1.301	0.227–7.447
Lesion length(mm)	0.037	0.024	2.515	1	0.113	1.038	0.991–1.087

CLTI = critical limb-threatening ischemia (statistically significant at *p* < 0.05); DCDs = drug-coated devices; BTK = below-the-knee; HR: hazard ratio (HR > 1 = increased risk of restenosis/occlusion; HR < 1 = protective effect); 95% CI: excludes 1.0 only for CLTI, confirming significance; * indicates statistically significant difference.

**Table 3:** Analysis of lesion types in non-CLTI vs. CLTI groups.

Variables	Non-CLTI (*n* = 49)	CLTI (*n* = 52)	Statistic	*p*-Value
Age (years)	72.08 ± 9.43	72.13 ± 8.48	*T* = −0.222	0.825
Male [*n*(%)]	41 (83.7%)	36 (69.2%)	*χ*² = 3.128	0.077
Follow-up duration (months)	16.57 ± 10.25	18.32 ± 9.74	*T* = −0.873	0.384
Comorbidities				
Hypertension [*n*(%)]	36 (73.5%)	47 (90.4%)	*χ*² = 5.375	0.020 *
Smoking [*n*(%)]	23 (46.9%)	26 (50.0%)	*χ*² = 0.105	0.746
Hyperlipidemia [*n*(%)]	35 (71.4%)	37 (71.2%)	*χ*² = 0.001	0.976
Chronic renal insufficiency [*n*(%)]	7 (14.3%)	3 (5.8%)	*χ*² = 1.943	0.163
Coronary heart disease [*n*(%)]	9 (18.4%)	12 (23.1%)	*χ*² = 0.375	0.54
Lesion length (cm)	24.29 ± 9.17	27.82 ± 9.52	*T* = −1.902	0.06
Device usage				
Debulking device [*n*(%)]	1 (2.0%)	2 (3.8%)	Fisher’s exact	1
DCD use [n(%)]	15 (30.6%)	25 (48.1%)	*χ*² = 3.629	0.057
Lesion types [*n*(%)]			*χ*² = 1.795	0.18
CTO lesions	19 (38.8%)	6 (11.5%)		
Stenosis	8 (16.3%)	4 (7.7%)		
Concurrent BTK outflow treatment [*n*(%)]	13 (26.5%)	18 (34.6%)	*χ*² = 0.889	0.346
Run-off [*n* (%)]			*χ*² = 4.223	0.121
1	18 (36.7%)	25 (48.1%)		
2	12 (24.5%)	17 (32.7%)		
3	19 (38.8%)	10 (19.2%)		
Total complications [*n*(%)]	0 (0%)	6 (11.5%)	Fisher’s exact	0.057
MALE	0	4		
Hematoma	0	1		
Cerebrovascular accident	0	1		

CLTI = critical limb-threatening ischemia; BTK = below-the-knee; DCDs = drug-coated devices; MALE = major amputation limb events; * indicates statistically significant difference.

## Data Availability

The data that support the findings of this study are available from the corresponding author upon reasonable request.
